# Generation of synthetic EEG data for training algorithms supporting the diagnosis of major depressive disorder

**DOI:** 10.3389/fnins.2023.1219133

**Published:** 2023-10-02

**Authors:** Friedrich Philipp Carrle, Yasmin Hollenbenders, Alexandra Reichenbach

**Affiliations:** ^1^Center for Machine Learning, Heilbronn University, Heilbronn, Germany; ^2^Medical Faculty Heidelberg, University of Heidelberg, Heidelberg, Germany

**Keywords:** major depressive disorder, electroencephalography, generative adversarial network, deep learning, data augmentation, synthetic data, biomarker, diagnosis

## Abstract

**Introduction:**

Major depressive disorder (MDD) is the most common mental disorder worldwide, leading to impairment in quality and independence of life. Electroencephalography (EEG) biomarkers processed with machine learning (ML) algorithms have been explored for objective diagnoses with promising results. However, the generalizability of those models, a prerequisite for clinical application, is restricted by small datasets. One approach to train ML models with good generalizability is complementing the original with synthetic data produced by generative algorithms. Another advantage of synthetic data is the possibility of publishing the data for other researchers without risking patient data privacy. Synthetic EEG time-series have not yet been generated for two clinical populations like MDD patients and healthy controls.

**Methods:**

We first reviewed 27 studies presenting EEG data augmentation with generative algorithms for classification tasks, like diagnosis, for the possibilities and shortcomings of recent methods. The subsequent empirical study generated EEG time-series based on two public datasets with 30/28 and 24/29 subjects (MDD/controls). To obtain baseline diagnostic accuracies, convolutional neural networks (CNN) were trained with time-series from each dataset. The data were synthesized with generative adversarial networks (GAN) consisting of CNNs. We evaluated the synthetic data qualitatively and quantitatively and finally used it for re-training the diagnostic model.

**Results:**

The reviewed studies improved their classification accuracies by between 1 and 40% with the synthetic data. Our own diagnostic accuracy improved up to 10% for one dataset but not significantly for the other. We found a rich repertoire of generative models in the reviewed literature, solving various technical issues. A major shortcoming in the field is the lack of meaningful evaluation metrics for synthetic data. The few studies analyzing the data in the frequency domain, including our own, show that only some features can be produced truthfully.

**Discussion:**

The systematic review combined with our own investigation provides an overview of the available methods for generating EEG data for a classification task, their possibilities, and shortcomings. The approach is promising and the technical basis is set. For a broad application of these techniques in neuroscience research or clinical application, the methods need fine-tuning facilitated by domain expertise in (clinical) EEG research.

## 1. Introduction

Major depressive disorder (MDD) is the most common mental disorder worldwide (World Health Organization, 2017) and characterized by episodes of mild to severe loss of motivation in various areas of life and cognitive deficits, leading to impairment in quality and independence of life (Otte et al., 2016). Even though systematic alterations in the affected organ, the brain, can be assessed quantitatively (Dev et al., 2022), MDD is routinely still diagnosed using interviews and questionnaires based on DSM-5 criteria (Bundesärztekammer (BÄK) et al., 2022). This approach is based on the patients’ symptoms, leading to diagnosis only after severe symptoms have already manifested and usually at least one acute phase has already been suffered (Zhang X. et al., 2022). Early diagnosis, however, can help the patient to receive prevention and early treatment to soften the disorder’s impact on the patient’s life (Habert et al., 2016). Clinicians have started complementing their diagnostic repertoire with electroencephalography (EEG) recordings, but to date, they still need the expertise and time to judge these recordings visually (Mahato and Paul, 2019). Therapy success is monitored using the same methods, leading to delayed detection of ineffective treatment (Zhang X. et al., 2022). In order to increase the sensitivity and objectivity of an MDD diagnosis, biomarkers based on neuroimaging have been explored in the last decade (Yasin et al., 2021; Dev et al., 2022).

The development of biomarkers based on machine learning (ML) methods applied to EEG data is a promising approach with diagnostic accuracies ranging from 70 to 99% (Yasin et al., 2021). The task of diagnosing a patient is here usually formulated as a classification problem, separating patients from healthy control (HC) subjects based on the EEG data. However, the generalizability of the results, as a basic requirement for clinical application, is often restricted by small datasets, leading to overfitting and, therewith, overestimating the diagnostic capability (Rakić et al., 2020). This is a common problem in the application of ML to clinical use cases since ML algorithms need large and diverse datasets to produce generalizable results with high fidelity. In particular, the latest generation of algorithms with deep learning (DL) that are well suited for complex problems like detecting small and distributed disease-induced changes in high-dimensional data like EEG are very data-greedy (Cho et al., 2015). The collection of clinical data, especially with recordings that are not routinely produced such as EEG in MDD patients, is time consuming and expensive. Furthermore, strict privacy policies in most countries protect patient data and prevent data sharing. Therefore, clinical datasets tend to be rather small from an ML perspective and/or only accessible to few researchers. One approach to train machine learning models with small datasets but still attain good generalizability is complementing the original data with artificially produced data (Nikolenko, 2019), a process termed data augmentation (DA). Data can be augmented by simple methods, such as the addition of noise or domain specific distortions, or more complex methods, like generating synthetic data with generative ML algorithms (Talavera et al., 2022). An additional advantage of synthetic data obtained with generative models is the possibility of publishing the data for other researchers to use to train their diagnostic models without the risk of violating patient data privacy.

Algorithms suitable for generating synthetic data that have been applied successfully to the creation of EEG data are generative adversarial networks (GAN), generative pre-trained transformers (GPT), and variational autoencoders (VAE) (Lashgari et al., 2020; He et al., 2021). GANs comprise two neural networks, a generator producing synthetic data from random noise and a discriminator judging whether the presented data is real or synthetic (Goodfellow et al., 2014). The training process gradually shifts the distribution of data produced by the generator toward the distribution of the real data. GPTs are models adapted from language understanding and production that learn the structure of arbitrary sequences and then synthesize the next data point in this sequence, therewith generating increasingly longer continuous data step-by-step (Radford et al., 2018). VAEs consist of an encoder network compressing the data into a low dimensional distribution from which the decoder network draws samples and expands them into the original data space, therewith generating data preserving the structure of the original data (Kingma and Welling, 2014). Even though the generation of synthetic time-series EEG data has been demonstrated successfully (Hartmann et al., 2018), the application for the clinical use case of augmenting EEG data for two clinical populations, patients and HC subjects, with synthetic data in order to train a “diagnosis classifier” is still sparse. Song et al. (2021) synthesized features derived from EEG for Alzheimer’s disease diagnosis with a GAN and demonstrated that they successfully generated data resembling patients and HC data distributions. Sobahi et al. (2022) constructed images from EEG features and created synthetic images with an extreme learning machine based autoencoder (ELM-AE). Augmenting the data with these images increased classification accuracy for schizophrenia diagnosis. Synthesizing time-series data from EEG directly has not yet been demonstrated for the clinical use case of a diagnosis classifier. However, this approach of generating the original data format from EEG for two clinical populations preserves most degrees of freedom for later data processing, e.g., for testing new biomarkers or publishing the data for further biomarker research.

In order to provide a comprehensive overview of current approaches for generating synthetic EEG data used for a classification task and an in-depth analysis of their advantages and potential pitfalls in a clinical use case, we first conducted a systematic review. In contrast to three previous reviews (Lashgari et al., 2020; He et al., 2021; Habashi et al., 2023), we focused on the clinical application of these methods rather than on the technical aspects. Therefore, we included studies that used all kinds of ML algorithms for classification instead of restricting the search to deep learning only. Most importantly, we focused on studies using generative methods only, i.e., creating truly synthetic data. This allowed for much deeper analyses of the methods specific to generative models and carves out the current shortcomings and next steps necessary specifically for the clinical use case of generating data for two or more clinical populations.

We conclude our work with an empirical study generating time-series EEG data for two clinical populations based on two publicly available datasets containing EEG data from MDD patients and HC (Mumtaz, 2016; Cai et al., 2020). For the generator and discriminator, we used convolutional neural networks (CNN) because of the complexity of the data. In order to improve the training stability of the discriminator, we adapted the Wasserstein GAN (WGAN) architecture (Arjovsky et al., 2017), frequently used for EEG data generation. In a WGAN, a *critic* minimizing the Wasserstein distance between real and synthetic data replaces the discriminator. For the generation of two datasets, MDD patients and HC, we adopted the frequently used conditional approach (Mirza and Osindero, 2014). For this approach, both the generator and critic get the label of the data, i.e., whether the data originated from a patient or HC, and the critic makes its judgment conditioned on this label. In order to judge the quality of the data, we first evaluated the signal qualities of the synthetic data qualitatively and quantitatively. Subsequently, we evaluated its usefulness by augmenting the real data for a diagnosis classifier. We compared the performance of the classifiers trained on the real data alone with the performance when trained on different ratios between real and synthetic data. Directly comparing the results based on two publicly available datasets allowed us to make conclusions about the generalizability of the findings and enables reproducibility.

## 2. Materials and methods

### 2.1. Systematic review

#### 2.1.1. Search strategy

**Figure 1:**
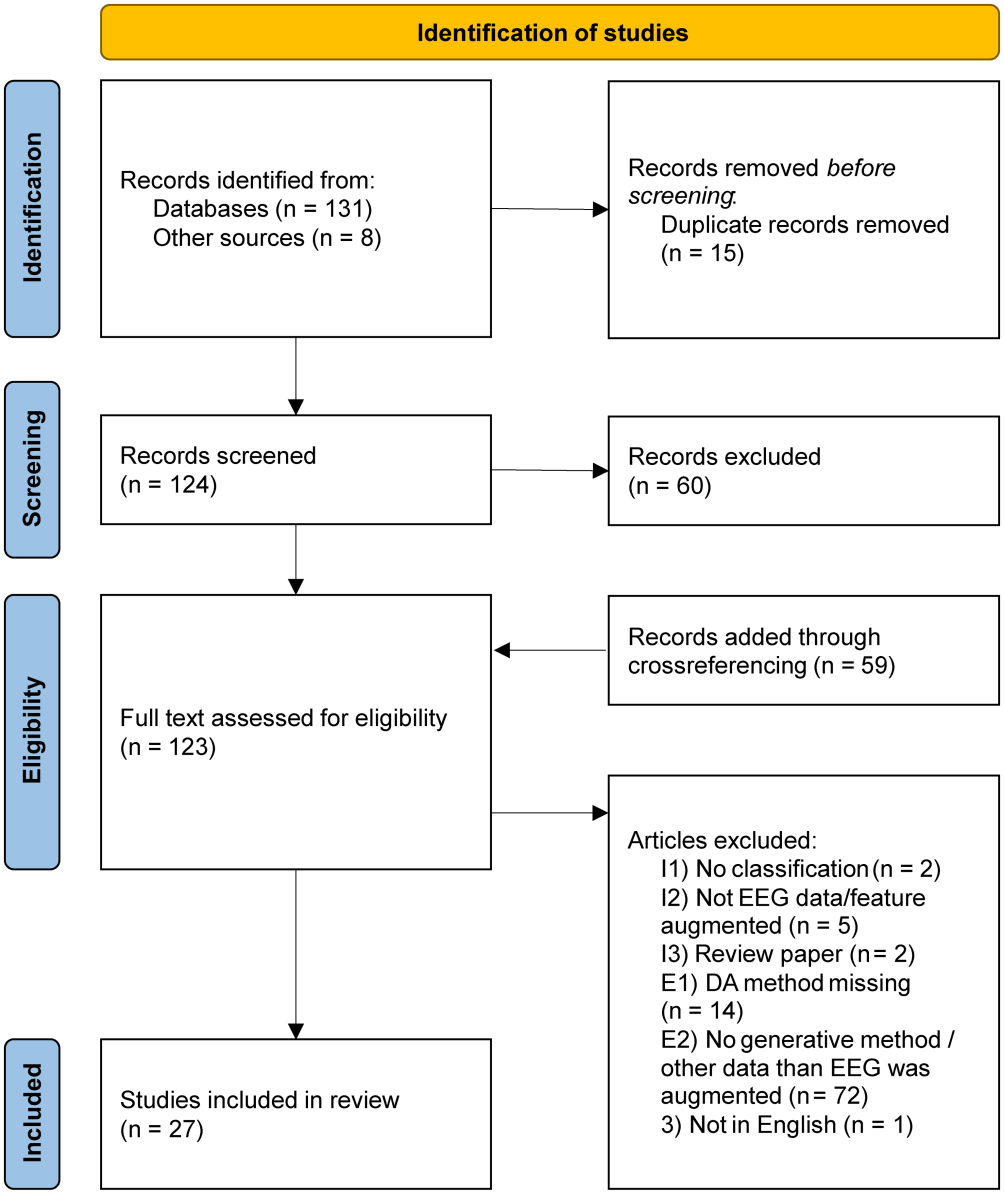
PRISMA flowchart for the paper search and selection process.

We conducted a systematic review according to PRISMA guidelines (Liberati et al., 2009) in the databases PubMed and IEEE Xplore on 12 August 2022 (Figure 1). The two databases were chosen to cover medical as well as technical literature. Originally, we were only interested in the clinical use case of generating synthetic EEG data for patients and their healthy counterparts in order to train a “diagnosis classifier” on the data. However, such clinical use cases were too rare, with only two studies found in the first search round. Therefore, we included any study that synthesized EEG data for a classification task. This included within-subjects studies with healthy volunteers performing some kind of cognitive task as well. We adapted the syntax of the two search strings for the respective databases and joined the results. Search strings: (1) “data augmentation” AND EEG AND diagnosis AND (ADHD OR Alzheimer OR dementia OR depression); (2) “data augmentation” AND EEG – only in abstract. We included the other diagnoses in the first search string because EEG-based biomarkers are suggested for these diseases as well (Leiser et al., 2011). After the removal of duplicates and papers after abstract screening, we added further papers based on cross-referencing. For full text assessment, three inclusion criteria were used: (1) EEG data were used for a classification task, (2) EEG data or features derived from EEG data were subjected to data augmentation, and (3) the studies were original research papers; and three exclusion criteria were used: (1) the data augmentation method was not specified, (2) there was no generative method for data augmentation, or other data than EEG data was generated, and (3) papers not published in the English language.

#### 2.1.2. Analysis

We aimed for a quantitative analysis of the aspects important for generating synthetic EEG data in a clinical use case. The **use case** *per se* informs whether the classification is conducted for a clinical purpose, about the paradigm used for data recording, and whether the experimental design was a within- or between-subjects design. A diagnosis classifier is always based on a between-subjects design. Regarding the input data, we needed to focus on the information specific for generating synthetic data. The **input/output** of the generator, i.e., whether it produces time-series or features and in which format, is reported in detail. However, the plethora of methodological details for EEG data recording and preprocessing is a general methodological issue when analyzing EEG data and would inflate the review. We, therefore, only comment on the breadth of methods here. The **generative model,** with its possible variants and their advantages and pitfalls, constitutes the core of the analysis. The next important item was the **evaluation** of the synthetic data with qualitative and quantitative methods. Finally, we investigated the **effect** of data augmentation on the original **classification task** with a special focus on the impact of the quantity of data generated.

### 2.2. Data augmentation

#### 2.2.1. Data

Two publicly available datasets were used for the empirical study (Mumtaz, 2016; Cai et al., 2020). Separately processing the datasets provided the possibility of direct replication of the results and therewith an account on the robustness of the methods. Both datasets contained 5-min resting-state EEG time-series from HC and MDD patients with eyes closed (Table 1). All patients were diagnosed based on the DSM-IV manual.

**Table 1:** Characteristics of the two publicly available datasets used for data augmentation.

	Dataset 1 (Cai et al., 2020)	Dataset 2 (Mumtaz, 2016)
# Subjects in dataset / after preprocessing	HC: 29/24 MDD: 24	HC: 28 MDD: 30/28
Medication	No	Unknown
Age [years] mean ± std	HC: 31.5 ± 9.2 MDD: 30.9 ± 10.4	HC: 38.2 ± 15.6 MDD: 40.3 ± 12.9
Location	Gansu Provincial Key Laboratory of Wearable Computing Lanzhou University, China	Hospital Universiti Sains Malaysia (HUSM) Malaysia
# Electrodes	128	19
Electrode referencing	Cz-referenced	Linked-ear-referenced
Sample frequency	250 Hz	256 Hz

For cleaning and preprocessing the EEG data, we used the Python toolbox *MNE* (Gramfort et al., 2014). To match the two datasets more closely, only the intersections of electrodes from both datasets were chosen, resulting in 13 channels: the frontal electrodes Fp1/2, F3/4, F7/8, and Fz, the central electrodes C3/4, parietal P3/4, and occipital O1/2. Both datasets were re-referenced to average (Yao et al., 2019). Preprocessing proceeded with band pass filtering (1 to 40 Hz) and automatic artifact removal with ICLabel (Li et al., 2022). One patient from dataset 2 was excluded because EEG was only recorded for 3 min. Both datasets were subsampled to the smaller class, with 24 and 28 subjects for each class, respectively.

Data were *z*-normalized per subject and channel separately. The time-series were then split into 8 s windows and outlier windows were removed. Any window with minimum or maximum values below or above 2* standard deviation of the average minimum or maximum values, respectively, was regarded as an outlier. The data of each window were then normalized between −1 and 1. The data resulting from the preprocessing are termed *real data* in the remainder of the article.

For dataset 1, the data frames used for both the classification and as input for the generative model were 2D matrices consisting of 13 (channels) rows and 250 (Hz) * 8 (s) = 2,000 columns. For dataset 2, respectively, the matrices had the shape (13, 256*8). For data generation, we used all available windows from the subjects to maximize the sample data. For the classification, we subsampled the number of windows to the respective smallest numbers of windows available, resulting in 18 windows per subject for dataset 1 and 16 windows for dataset 2. The channels were ordered based on head topology with the left hemisphere electrodes first (Fp1, F3, C3, P3, O1, and F7), then the central (Fz), and finally the corresponding right ones (Fp2, F4, C4, P4, O2, and F8).

#### 2.2.2. Data generation

The baseline for data augmentation not using generative methods was obtained with **noise addition** (Yang et al., 2023), the simplest and most frequently used method for generating artificial EEG data without a generative approach (Lashgari et al., 2020). We added uniform noise between −0.1 and + 0.1, corresponding to 10% of the normalized original signal amplitude, to the preprocessed time-series signal. After noise addition, the data was again normalized between −1 and 1. The data resulting from this procedure are termed *noise data* in the remainder of the paper.

The **generative method** for creating the *synthetic data* was a conditional Wasserstein GAN with a generator and critic consisting of CNNs loosely adapted from the work of Panwar et al. (2019, 2020) and optimized for our use case (for the detailed architectures, *cf.* Appendix Tables A1, A2). The generator input was a latent vector of size 100 initialized from a standard normal distribution. The input was reshaped to represent the channels in one and discrete sample times in the other dimension. The generator had four transposed convolutional layers that upsample and resize and one convolutional layer that only resizes the input. In between those layers, Leaky Rectified Linear Unit (ReLU) activation and batch normalization were used. The final output had the same shape as the real data. The activation function in the last layer was the hyperbolic tangent to obtain values ranging from −1 to 1 analog to the real data.

The input layer of the critic had the same shape as the real data and the generator output. Before it was downsampled, a Gaussian noise layer augmented the real and synthetic data to prevent the critic from memorizing the real data, which is likely to happen for small datasets (Zhao et al., 2020). Afterward, the critic reduced the dimension of the input data with two convolutional layers featuring strides of (2, 2) and a third convolutional layer featuring a stride of (1, 2). In between those layers, Leaky ReLU activation was used. The final two layers were a dropout layer to prevent overfitting (Lee and Lee, 2020) and a dense layer with linear activation returning the critic score. The Wasserstein distance was used as a loss function (Rüschendorf, 1985) with weight clipping. For the optimizer, the Adam algorithm (Kingma and Ba, 2014) was chosen with a learning rate of 0.0005, β1 = 0.0, β2 = 0.999, and ε = 10–7 (Hartmann et al., 2018). During training of the generator and critic, the latter was trained for five iterations for each iteration of the generator, as proposed in the original Wasserstein paper (Arjovsky et al., 2017). This helps the critic to detect poorly augmented data more easily.

#### 2.2.3. Diagnosis/classification

For classification of the real data and evaluation of the augmented data, the preprocessed and/or generated EEG time-series windows were subjected to a CNN with an architecture adapted from *DeprNet* (Seal et al., 2021). The network consists of five convolutional, max-pooling, and batch normalization layers each, followed by three fully connected layers. *DeprNet* was optimized for the diagnosis of MDD from time-series EEG data and can therefore be utilized with only small modifications to accommodate the difference in number of channels, window size, and sample frequency. For the last fully connected layer, we implemented a sigmoid activation function because pretests yielded better results than the original softmax function. The initial parameters for *DeprNet* were chosen based on the values from the original study: binary cross entropy as loss function; Adam optimizer (Kingma and Ba, 2014) with learning rate 0.0005, β1 = 0.9, β2 = 0.999, and ε = 10–7; classification accuracy as evaluation metric. The classification accuracies with their confidence estimated were obtained with leave-two-subjects-out cross-validation, i.e., each test fold included one HC and one MDD. This procedure resulted in subject-wise cross-validation (Saeb et al., 2017).

#### 2.2.4. Evaluation of the synthetic data

First, generated time-series data samples and their spectra were inspected visually. We chose the frontal electrodes as examples because abnormalities in frontal electrodes are frequently reported for MDD (Stewart et al., 2010). The exemplary single time-series from electrodes F3 and F4 served as the visual impression of the smoothness and form of the signal, while the mean time-series signals and their 95% confidence interval across subjects gave an impression of the general distribution of the continuous data over time. For the mean signals, we randomly chose one window from ten randomly chosen subjects and ten random synthetic data windows.

The frequency spectra based on the periodograms of the synthetic data reveal how well the generated signals resemble the real data in the frequency domain. Here, we only present mean and 95% confidence intervals across subjects because this data can be averaged meaningfully, resulting in an estimate of the population’s spectrum. For each subject, we calculated the mean across all windows as a robust individual estimate and then matched the number of synthetic data frames with the number of subjects from each clinical group and dataset. For a quantitative assessment of commonalities and differences between spectra of real and synthetic data, we also present the averages of the commonly used frequency bands delta (0.3–4 Hz), theta (4–8 Hz), alpha (8–12 Hz), and beta (12–30 Hz). Analyses of variance (ANOVA) within datasets and frequency bands with between-subjects factors data type (real/synthetic) and diagnosis (HC/MDD) further qualify whether differences between real and synthetic data predominate or whether differences between diagnostic groups outweigh these.

Finally, the most important metric for the synthetic data was the performance of the classifier diagnosing either based on the synthetic data only or on combinations of real and synthetic data. Four classifiers were **trained** for both types of data augmentation, and all were **tested** on real data only (Table 2). Note that the first two classifiers with augmented data were trained on the same amount of data as the classifier trained on the real data only (Table 2, first row). The two remaining classifiers were trained on two or three times as much data, respectively.

**Table 2:** Number of data frames used for each fold of the classifiers.

	Dataset 1		Dataset 2	
	Train data	Test data	Train data	Test data
Real data	13 × 46 × 18	13 × 2 × 18	13 × 54 × 16	13 × 2 × 16
Noise data or synthetic data	13 × 46 × 18	13 × 2 × 18	13 × 54 × 16	13 × 2 × 16
50% real + 50% noise/synt	13 × (23 + 23) × 18	13 × 2 × 18	13 × (27 + 27) × 16	13 × 2 × 16
100% real +100% noise/synt	13 × (46 + 46) × 18	13 × 2 × 18	13 × (54 + 54) × 16	13 × 2 × 16
100% real +200% noise/synt	13 × (46 + 92) × 18	13 × 2 × 18	13 × (54 + 108) × 16	13 × 2 × 16

The numbers result from number of electrodes (13) × 2*number of “subjects” × number of windows per “subject”.

The performance of the classifiers was compared using one-sided t-tests with *p* = 0.05, not corrected for multiple comparisons, considered significant. The classifiers trained on the synthetic data and the ones trained on the combination of real and synthetic data were expected to perform better than the classifiers based on the real data alone and better than the respective classifier trained on noise data. All reported results are mean values with 95% confidence intervals unless stated otherwise.

## 3. Results

### 3.1. Systematic review

The database search yielded 27 papers eligible for this review (Figure 1). The first paper appeared in 2018 (Hartmann et al., 2018).

#### 3.1.1. Use case for classification and EEG paradigm

**Figure 2:**
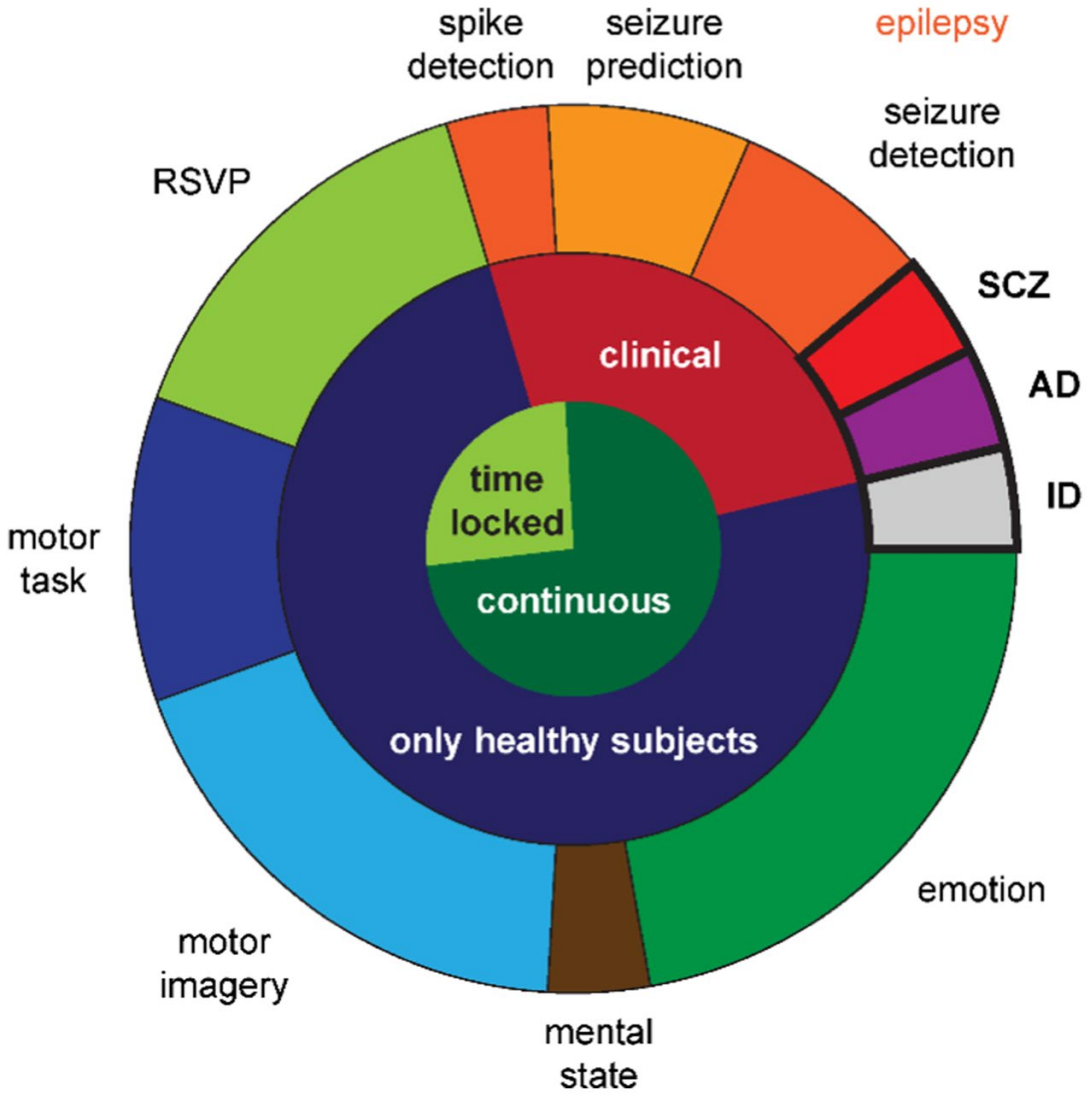
Distribution of use cases across studies. The inner circle denotes whether the EEG time-series is locked to an event or continuous. The middle ring denotes whether the classification serves a clinical purpose. The outer ring details the specific use cases with the between-subjects class designs emphasized with bold outlines and font. Note that the shades of orange further differentiate the epilepsy use cases into three types of classification goals. RSVP: rapid serial visual presentation; SCZ: schizophrenia; AD: Alzheimer’s disease; ID: identification.

Only two studies reported the **clinical use case** of supporting the diagnosis of a psychiatric or neurodegenerative disease: diagnosing Alzheimer’s disease (Song et al., 2021) or schizophrenia (Sobahi et al., 2022) (*cf.* Figure 2, violet and red segment in the outer ring). Five more studies in the clinical field (*cf.* Figure 2, red segment in the middle ring) revolved around epilepsy (*cf.* Figure 2, orange segments in the outer ring). Two studies aimed to detect an ongoing seizure or its onset (Haradal et al., 2018; Wei et al., 2019), two studies aimed to predict an upcoming seizure (Niu et al., 2021; Rasheed et al., 2021), and the last one aimed at detecting spikes occurring between seizures (Geng and Chen, 2021). Note that all five epilepsy studies had a within-subjects design, i.e., all patients contributed data for all classes.

The third and last **between-subjects study** used EEG data for identifying a person, i.e., biometric identification (Piplani et al., 2018) (*cf.* Figure 2, gray segment in the outer ring). This study and the remaining ones, using behavioral paradigms to elicit different cognitive states that were then classified, collected data from healthy subjects only (*cf.* Figure 2, dark blue segment in the middle ring).

The nature of the EEG time-series in the four studies utilizing the rapid serial visual presentation (RSVP) paradigm (Panwar et al., 2019, 2020; Xu et al., 2022; Zhang R. et al., 2022) (*cf.* Figure 2, light green segment in the outer ring) differed to most other EEG recordings in the reviewed studies with respect to the continuity in the signal. This was a **time locked paradigm** (*cf.* Figure 2, light green segment in the inner ring) assessing the P300 component of visual evoked potentials. The only other studies with time locked data were the epilepsy study for spike detection and two of the motor task studies (Abdelfattah et al., 2018; Fahimi et al., 2021) (*cf.* Figure 2, dark blue segment in the outer ring). The remaining study with a motor task recorded continuous EEG data during left hand movement vs. rest (Hartmann et al., 2018).

The remaining 12 studies generated data based on **continuous EEG data** (*cf.* Figure 2, dark green segment in the inner ring) during **cognitive tasks**. Bird et al. (2021) elicited the three mental states relaxed, neutral, and concentrated, which they later classified (*cf.* Figure 2, brown segment in the outer ring). The five studies in the field of motor imagery (Ko et al., 2019; Yang et al., 2019; Zhang et al., 2020, 2021; *cf.* Figure 2, light blue segment in the outer ring) all used public data provided for the brain–computer-interface (BCI) competitions (Sajda et al., 2003): BCI competition II dataset 3 with left and right hand movements (Schlögl et al., 1997) and BCI competition IV datasets 1 with two out of left hand, right hand, or foot movements (Blankertz et al., 2007), 2a with left and right hand, feet, and tongue movements (Naeem et al., 2006), and 2b with left and right hand movements (Leeb et al., 2007). From the six studies in the field of emotion recognition (*cf.* Figure 2, dark green segment in the outer ring), one recorded their own data with positive and negative emotions (Chang and Jun, 2019), while the remaining five (Luo and Lu, 2018; Luo et al., 2019, 2020; Pan and Zheng, 2021; Kalashami et al., 2022) used the publicly available datasets Database for Emotion Analysis using Physiological Signals (DEAP) with the two orthogonal dimensions valence and arousal, allowing for two different two-class classifiers or a four-class classifier (Koelstra et al., 2011), SJTU Emotion EEG Dataset (SEED) with positive, neutral, and negative emotions (Zheng and Lu, 2015), and SEED-V with the five emotions happiness, sadness, disgust, neutral, and fear (Liu et al., 2021).

Altogether, 18 studies used publicly available datasets and did not record their own data.

#### 3.1.2. Input/output data

For recording and preprocessing EEG data, a wide variety of methods exists (Robbins et al., 2020). Since these differences are common to all EEG analyses and not just data synthetization, we only comment on the variety but refrain from analyzing them in depth in order to keep the review concise. Data were recorded from three to roughly 100 subjects using one to 256 EEG channels in different sampling frequencies. Common preprocessing steps included re-sampling, filtering, artifact removal, normalization, and splitting the EEG time-series into overlapping or non-overlapping windows.

The data format finally fed into the generative model equals the format of the generated data. The majority of studies (*n* = 15) used **time-series data**. Generating raw time-series provides the highest degree of freedom for processing the synthetic data afterward. Six studies used 2D matrices with time (samples) in the one dimension and location (channels) in the other dimension, similar to our study. Five further studies used 1D vectors in the time dimension: two studies used data from only one channel overall, two studies based on intracranial EEG used the time-series independent of the recording electrode, and the last study modeled each channel independently. In the remaining four studies, we were not able to determine the detailed format of the input data. All seven studies with a time locked paradigm used time-series data; the remaining eight used continuous EEG like our study.

From the remaining studies, seven calculated **features** from the time-series used as 1D input vectors for data generation. The most common features were differential entropy (DE) and/or power spectral density (PSD) for delta (if possible), theta, alpha, beta, and gamma bands (n = 4). One study used the raw power spectrum and the remaining two utilized more complex sets of features that had been developed for previous studies. The study on Alzheimer’s diagnosis (Song et al., 2021) belongs to the latter.

From the five studies that converted their time-series to **images**, the majority (n = 4) used time frequency representation (TFR), either one image per channel or channels stacked in the frequency domain. The remaining study was the one conducting the Schizophrenia diagnosis (Sobahi et al., 2022) and constructed an image from frequency features.

Due to conflicting information in the papers, there is some uncertainty in the assignment of two studies.

#### 3.1.3. Generative model

The most popular model for generating synthetic EEG data for a classification task was by far the GAN (*n* = 24, Table 3). Only two studies adapted the GPT principle from language processing, and we found one autoencoder that was used in its own right and not just as comparison for a GAN-based approach. When several generative algorithms were compared in a study, we only extracted the one with the best result. For generating labeled data, i.e., distinct data for each class, five approaches were applied. The most popular was the conditioning of generator and discriminator with the class label (*n* = 10). The intuitive approach of simply generating the data of each class separately was adopted in seven studies. In six studies, the GAN was used for boosting the minority class, hence only the minority class was generated. The auxiliary discriminator that feeds the result of the data classification in generator and discriminator learning as well was used in three studies. The last study generated one distribution from all data and assigned class labels posthoc based on a classifier trained on the real data.

**Table 3:** Overview of generative models and their frequency of use.

	Class differentiation:	Only minority	1 Generator/class	Conditional	Auxiliary	Posthoc selection	Total
GAN	CNN total (WD, GP, select)	4 (2, 2, 1)	1	5 (2, 2, 0)	1 (1, 1, 0)	1 (1, 1, 1)	**12** **(6, 6, 2)**
	MLP total (WD, GP, select)	2 (1, 0, 0)	1	5 (4, 2, 3)			**8** **(5, 2, 3)**
	RNN		2		2		**4**
GPT			2				**2**
AE			1				**1**
**Total**		**6**	**7**	**10**	**3**	**1**	***27***

The GAN models are further differentiated by the network making up the generator and discriminator. The five ways to generate class-specific data (columns) are explained further in the text. The number of studies improving their training with the Wasserstein distance and/or gradient-penalty are added, as well as the number of studies using selective augmentation. GAN: generative adversarial network; GPT: generative pre-trained transformer; AE: autoencoder; CNN: convolutional neural network; MLP: multi-layer perceptron; RNN: recurrent neural network; WD: Wasserstein distance; GP: gradient penalty.

##### 3.1.3.1. Generative adversarial network

**Figure 3:**
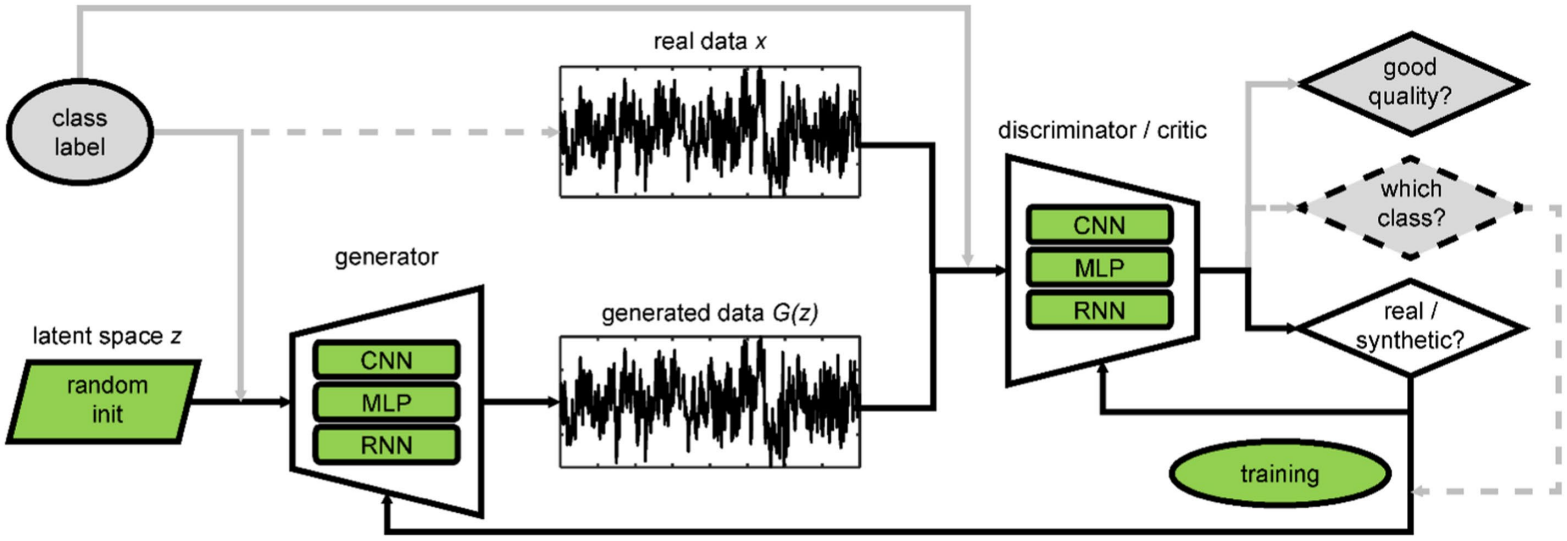
Overview of GAN architectures with variants (green) and optional elements (gray). The dashed elements belong to the GAN with auxiliary classification. Please refer to the text for details. CNN: convolutional neural network; MLP: multi-layer perceptron; RNN: recurrent neural network.

The first proposed architecture of a GAN (Goodfellow et al., 2014), often referred to as *Vanilla GAN*, comprises two multi-layer perceptrons (MLP), the generator and discriminator, competing against each other (*cf.* Figure 3). The generator transforms values *z* randomly drawn from a standard normal distribution into synthetic data *G(z)*. The goal of the generator is to generate data the discriminator cannot distinguish from the real data *x*. With training, the discriminator maximizes its loss while the generator minimizes its loss using the Jensen–Shannon divergence between real and synthetic data distributions in the case of the Vanilla GAN.

**Figure 4:**
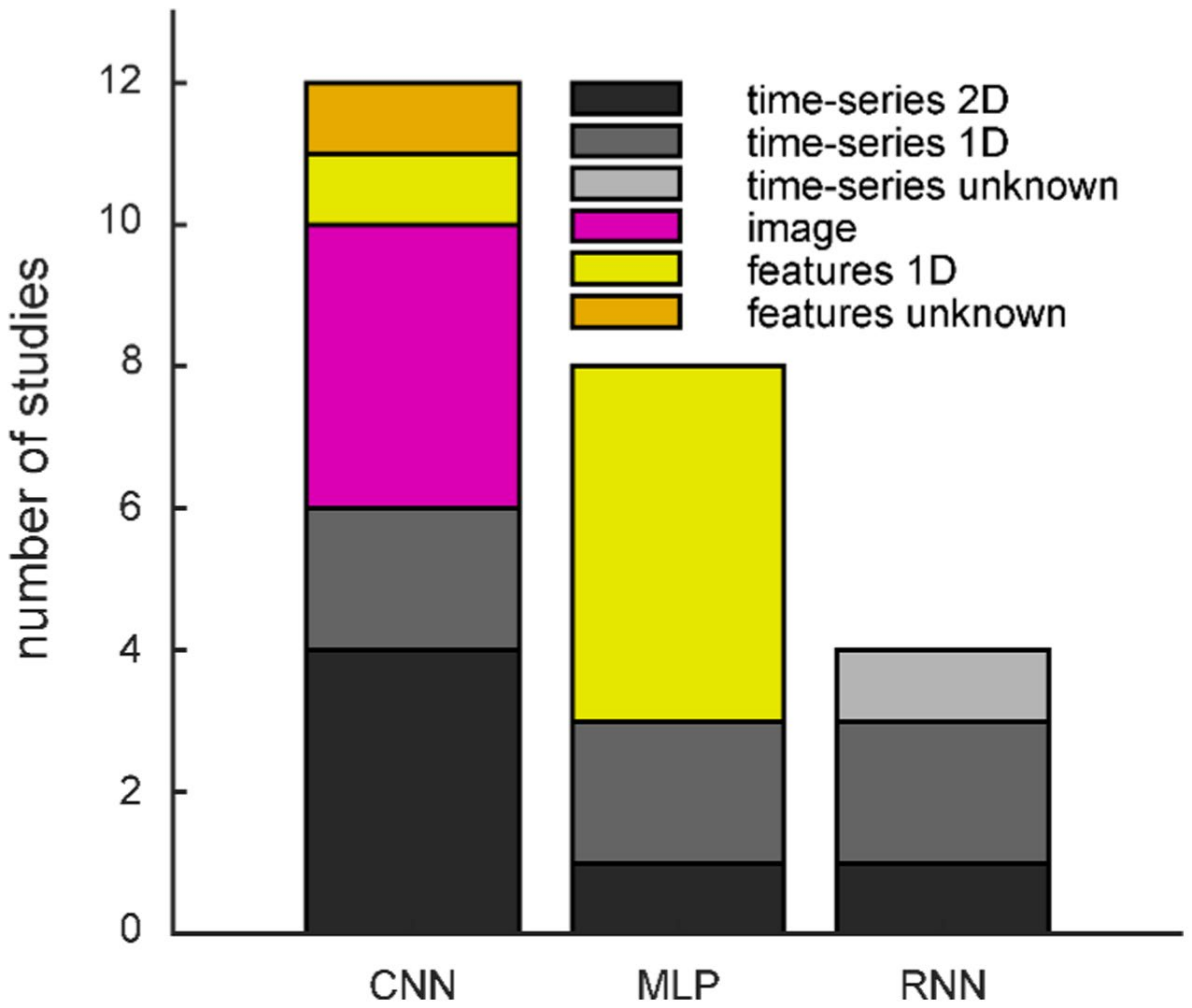
Frequency of generator/discriminator networks split by input/output formats. CNN: convolutional neural network; MLP: multi-layer perceptron; RNN: recurrent neural network.

Common variations of a GAN feature another network architecture for the generator and/or discriminator (*cf.* Table 3 and Figure 3 green rounded boxes). CNNs are a popular choice due to their hierarchical structure, successively combining groups of local data points, which resembles neural organization principles and makes them well suited for processing biological data (LeCun et al., 2015). When a CNN architecture is used, the input is often organized in two dimensions, and all studies feeding images into the GAN use CNNs (Figure 4). Note that the spatial neighborhood plays a critical role in CNN architectures since neighboring data points are combined *via* the convolution layers. The Vanilla GAN uses an MLP, and this architecture is still a popular choice. However, especially for this category, the detailed network architecture was often hard to determine, and we included a study in this category when the architecture was called a neural network or deep neural network (DNN), but the description did not sound like a CNN or recurrent neural network (RNN). Therefore, networks with one or more hidden layers were subsumed in this category. Most studies using MLP feed a 1D vector as input (Figure 4). Using an RNN as generator is motivated by the inherent properties of RNNs to deal with time dependencies, and the EEG signal has that property (Abdelfattah et al., 2018). Consequently, all studies utilizing an RNN fed time-series data into the GAN (Figure 4).

The next variations address the instabilities in training (*cf.* Figure 3 green ellipse) sometimes encountered with the Vanilla GAN. Mode collapse occurs when the generator produces very small variations of the data because only those are recognized as real data by the discriminator (Saatci and Wilson, 2017). This problem is overcome by using a different loss function; the most popular choice here is the Wasserstein distance (WD, Table 3) (Arjovsky et al., 2017), sometimes combined with gradient penalty (GP) (Gulrajani et al., 2017) to enforce Lipschitz continuity instead of weight clipping. In a Wasserstein GAN, the discriminator is called the critic. Along with using the WD, Arjovsky et al. (2017) also proposed a training regimen in which the critic iterates several times before the generator runs again, leading to even more stability in training. This adaptation can be observed in some of the studies as well. Other modifications observed are (1) gradually increasing data resolution with training (Hartmann et al., 2018), (2) augmenting the synthetic and real data with a Gaussian noise layer before feeding them into the discriminator (Zhao et al., 2020), or (3) initializing the weights of the generator and discriminator with the weights of the decoder and encoder of a VAE (Xu et al., 2022).

Most often, the generator is initialized with random uniform noise, but we also found variations using, for example, Gaussian noise (*cf.* Figure 3 green parallelogram) (Yang et al., 2019). For the last step in the process, there is an optional addition selecting only good quality data for the synthetic dataset (Table 3, Figure 3 top right rhombus).

Finally, two approaches extend the GAN architecture to generate more than one class of data (Table 3, Figure 3). The most popular method is conditioning the generator and discriminator with the class label (Mirza and Osindero, 2014). In contrast, the auxiliary approach feeds the result of a data-label classification into the training process in addition to the results from the real vs. synthetic classification. The reviewed studies achieved the data-label classification by extending the architecture of the existing discriminator (*cf.* Figure 3 dashed elements) (Panwar et al., 2020; Geng and Chen, 2021). Note that other methods exist, e.g., utilizing an extra classifier that gets only the generated data as input (Liao and Dong, 2022).

Some authors give the variation of their GAN acronyms according to the aspect that is important to them. However, the acronyms often do not fully describe the architecture. E.g., a GAN with a CNN using the Wasserstein distance may be referred to as DCGAN, emphasizing the deep convolutional architecture of the generator and discriminator. Alternatively, it may be called WGAN, emphasizing the training based on the Wasserstein distance. Other common acronyms are RGAN for using RNN in the generator, WGAN-GP for the Wasserstein GAN using gradient penalty, cGAN for conditional GANs, or AC-GAN for GANs with auxiliary classifiers.

##### 3.1.3.2. Generative pre-trained transformer

The two studies using GPT models (Bird et al., 2021; Niu et al., 2021) based their architecture on GPT-2 (Radford et al., 2018) trained on natural language from millions of websites. Both used continuous time-series data (data format not otherwise specified) as input.

##### 3.1.3.3. Autoencoders

Only one study utilized a variant of the autoencoder as only generative model (Sobahi et al., 2022). They used an extreme learning machine based autoencoder (ELM-AE) on an image constructed from frequency features. Four more studies (Luo et al., 2020; Zhang et al., 2020; Fahimi et al., 2021; Song et al., 2021) compared their GAN architectures against the performance of data generated with a VAE but found the GAN results superior.

#### 3.1.4. Evaluation metrics and methods

The purpose of the synthetic data of all reviewed papers was to improve the training of a classifier. However, the good quality of the generated data is a prerequisite for a meaningful improvement of the classification. Furthermore, the stability of the training process might be of interest in some cases as well. Nonetheless, seven papers did not perform any kind of evaluation. Seven papers evaluated only **training stability** quantitatively to demonstrate the presence or absence of convergence failure (Zhang et al., 2018) or mode collapse by showing or describing generator and/or discriminator loss curves or discriminator accuracy. For two of those studies, this was the only evaluation at all.

The **quantitative evaluation metrics** for the generated data were rather sparse and scattered. They fall into roughly two categories: the first judges the similarity between time-series, or their respective diversity, based on cross-correlation (n = 1) or Euclidian distance (n = 1), or, in the case of time locked data, with dynamic time warping (DTW) with Manhattan distance (n = 1). The second provides metrics for describing the distances between the data distributions either using an inception classifier and reporting Fréchet inception distance (FID) (Heusel et al., 2017) (*n* = 2) or inception score (IS) (Salimans et al., 2016) (*n* = 1), or the Gaussian mixture model (GMM) log-likelihood distance (*n* = 2), maximum mean discrepancy (MMD) (*n* = 1), Kullback–Leibler (KL) divergence (*n* = 1), or sliced Wasserstein distance (Peyré and Cuturi, 2017) (*n* = 1). One paper reported the reconstruction accuracy of the signal.

**Figure 5:**
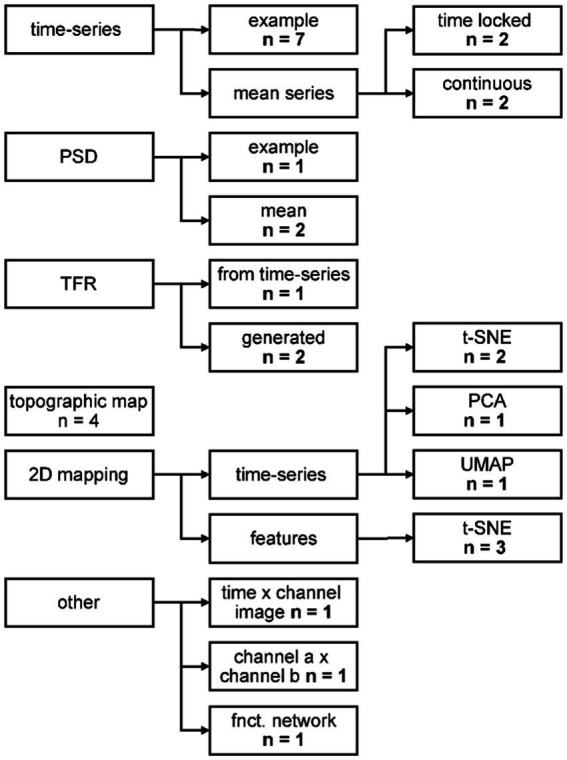
Categorization of the qualitative evaluation methods. PSD: power spectral density; TFR: time frequency representation; t-SNE: *t*-distributed Stochastic Neighbor Embedding; PCA: principal component analysis; UMAP: uniform manifold approximation and projection. For further description, see text.

Thirteen out of 18 papers, presenting **qualitative evaluation** of the generated data, provided them in addition to quantitative metrics. The most common visual representation for qualitative assessment of the synthetic data were figures of exemplary single time-series data (Figure 5). Presenting the mean of the time-series serves two purposes, dependent on the type of data: for time locked paradigms, the mean time-series reveals whether the expected response shape is present in the synthetic data. For continuous paradigms, the mean time-series shows the distribution at each time point of the arbitrary frames, and one might detect systematic differences between the time-series. Transforming data from time to frequency space and showing frequency spectra reveals whether the frequency content of the original signal is captured properly. The power spectral density (PSD) was shown in three studies. Three more studies illustrated the data in time frequency representation (TFR), with two of them generating the data already in this format and the third performing the transformation for visual inspection only. Topographic maps are a common way of depicting EEG data and were used for visual inspection by four studies. A 2D mapping of the generated data by various algorithms (*cf.* Figure 5) provides an overview of whether the distribution of the synthetic data matches the real data and was conducted by seven studies overall. Three studies had individual visual representations for their data (*cf.* Figure 5 “other”).

From the eight studies using continuous EEG time-series as input/output like our study, three did not perform any kind of evaluation, and one study presented only generator and discriminator loss. The remaining four studies all showed single exemplary time-series, and two transformed the data to frequency space for a visual comparison (Hartmann et al., 2018; Bird et al., 2021). Only one study provided quantitative metrics with FID, IS, Euclidean, and sliced Wasserstein distances (Hartmann et al., 2018).

#### 3.1.5. Effect on classification

Since the data was generated to improve a classification task, most studies trained one or several classification algorithms with the augmented data to demonstrate the effect of the augmentation on the classification. The manifold of ML algorithms used for classifying EEG data is not the focus of this review, therefore, we only provide a brief summary here. Eighteen studies used some kind of DL algorithm directly on the data generated, with the CNN being the most popular by far. Ten studies used classical ML approaches, mainly support vector machine (SVM) but also a variety of decision trees or occasionally other algorithms. Five of those studies applied the classifiers on the features that originated directly from the generative model. The remaining five studies generated time-series and two of them used the time-series data for classification as well. The other three studies calculated statistical, event-related potential (ERP), or connectivity features just for the classification.

##### 3.1.5.1. Overview of effects

**Figure 6:**
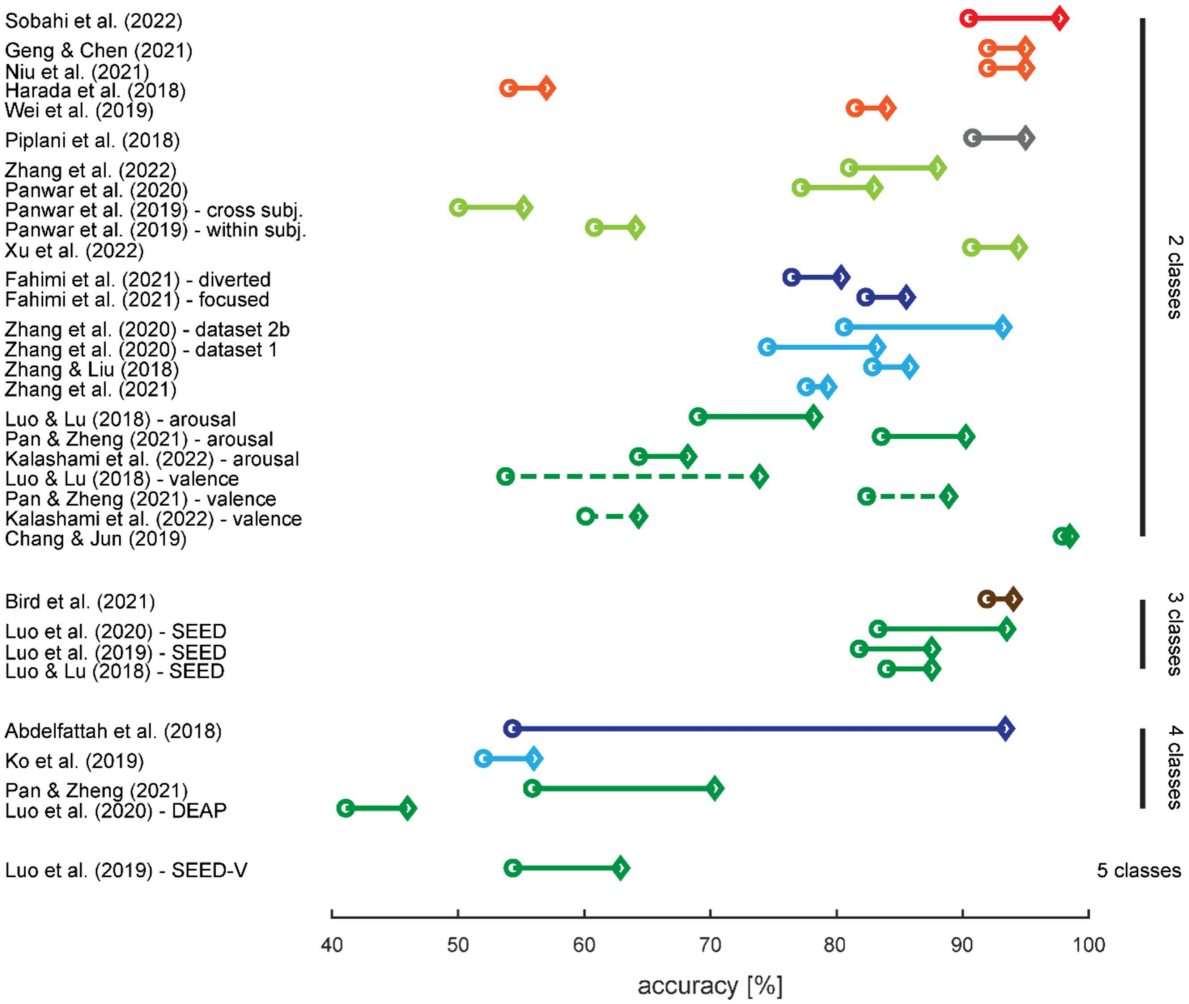
Effect of augmentation with synthetic data on classification performance. The circles denote the classification accuracies with training on real data only, and the diamonds denote the highest increase in accuracy with training on augmented data. Results are sorted first by number of classes in the classification task, then by use case (*cf.* color coding), and finally from the highest to lowest increase in accuracy. When more than one classification task was tested in a study, the results were split up. The colors are adopted from Figure 2; a dashed line differentiates two classification tasks for the same number of classes within a study.

For an overview of the effect of augmenting data with synthetic data, we first extracted the highest effect in terms of absolute accuracy increase from each study and classification task (Table 4, Figure 6). When accuracies were only depicted in figures in the original paper, we estimated them visually.

**Table 4:** Mean baseline accuracies and their improvements with augmented data for classifiers with two to five classes.

Number of classes	2	3	4	5
*n*	24	4	4	1
Baseline accuracy [%]	76.91 ± 5.41	85.26 ± 4.45	51.22 ± 5.78	54.34
Accuracy improvement [%]	5.51 ± 1.65	5.41 ± 3.46	15.62 ± 16.03	8.53

A high baseline classification accuracy, i.e., classification performance for training with real data only, provides limited possibilities for improvement; therefore, we expected the highest increase for studies with low baseline accuracies. This is not immediately obvious from Figure 6 but we did indeed find a small negative correlation between baseline accuracy and the amount of accuracy increase when training on augmented data (*r* = −0.37; *t*_31_ = −2.249; *p* = 0.032).

##### 3.1.5.2. Effect dependent on the amount of generated data

**Figure 7:**
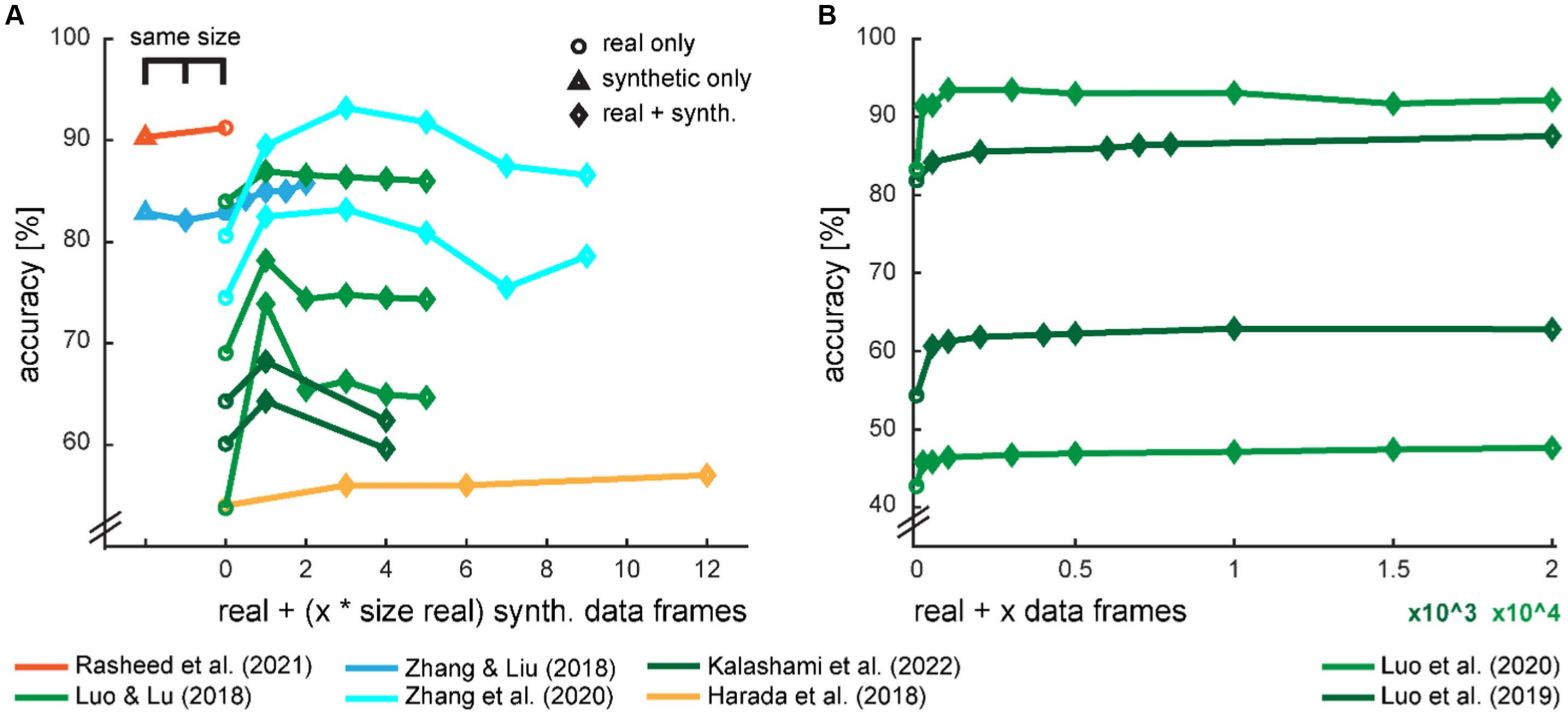
Development of classification accuracy with the ratio of real and synthetic data. The amount of synthetic data in the augmented dataset was either provided in multiples of the size of the real training dataset **(A)** or in the absolute amount of synthetic data frames **(B)**. Two studies also provide a comparison with the equal amount of training data, either with a fully synthetic dataset or a mixed set with half real and half synthetic data. The color scheme is loosely adopted from Figure 2.

Some studies provide data on the accuracy development dependent on the amount of generated data for training the classifier (Figure 7). On an intuitive notion, the classification accuracy should increase with increasing size of training data and eventually level out when the generated data cannot provide additional information. However, we saw in several studies that there seems to be an optimal amount of additional synthetic data, and the accuracies drop with even more data.

#### 3.1.6. Publication

Fourteen studies from this review were journal articles, mainly published *via* IEEE Xplore (IEEE, New York, NY, United States) (*n* = 7), otherwise from Hindawi (Hindawi Limited, London, United Kingdom) with two articles, and one each from Elsevier (Elsevier B.V., Amsterdam, Netherlands), IOPscience (IOP Publishing, Bristol, United Kingdom), MDPI (MDPI AG, Basel, Switzerland), and Taylor & Francis (Taylor & Francis Groups, Abingdon, United Kingdom). Eight of these articles were published in journals that are dedicated to methods in biology, medicine, or neuroscience or interdisciplinary journals comprising one of these disciplines and computation or engineering on the other side. One article was published in a journal specialized for architecture and building engineering. The remaining five articles were published in journals in the fields of computer science or engineering.

Eleven articles were full conference papers, also mainly published *via* IEEE Xplore (*n* = 9), as well as one each from ACM Digital Library (ACM, New York, NY, United States) and Springer (Springer-Verlag GmbH, Berlin, Germany). Five articles were presented at conferences dedicated to methods in biology, medicine, or neuroscience, with the remaining six at computer science or engineering conferences.

Two of the articles included in the review were preprints accessed from arXiv.

Ten articles had gaps in the methods that make them non-reproducible. These include missing information on the data source, preprocessing, data synthetization, and/or data evaluation.

### 3.2. Data augmentation

#### 3.2.1. Synthetic data

**Figure 8:**
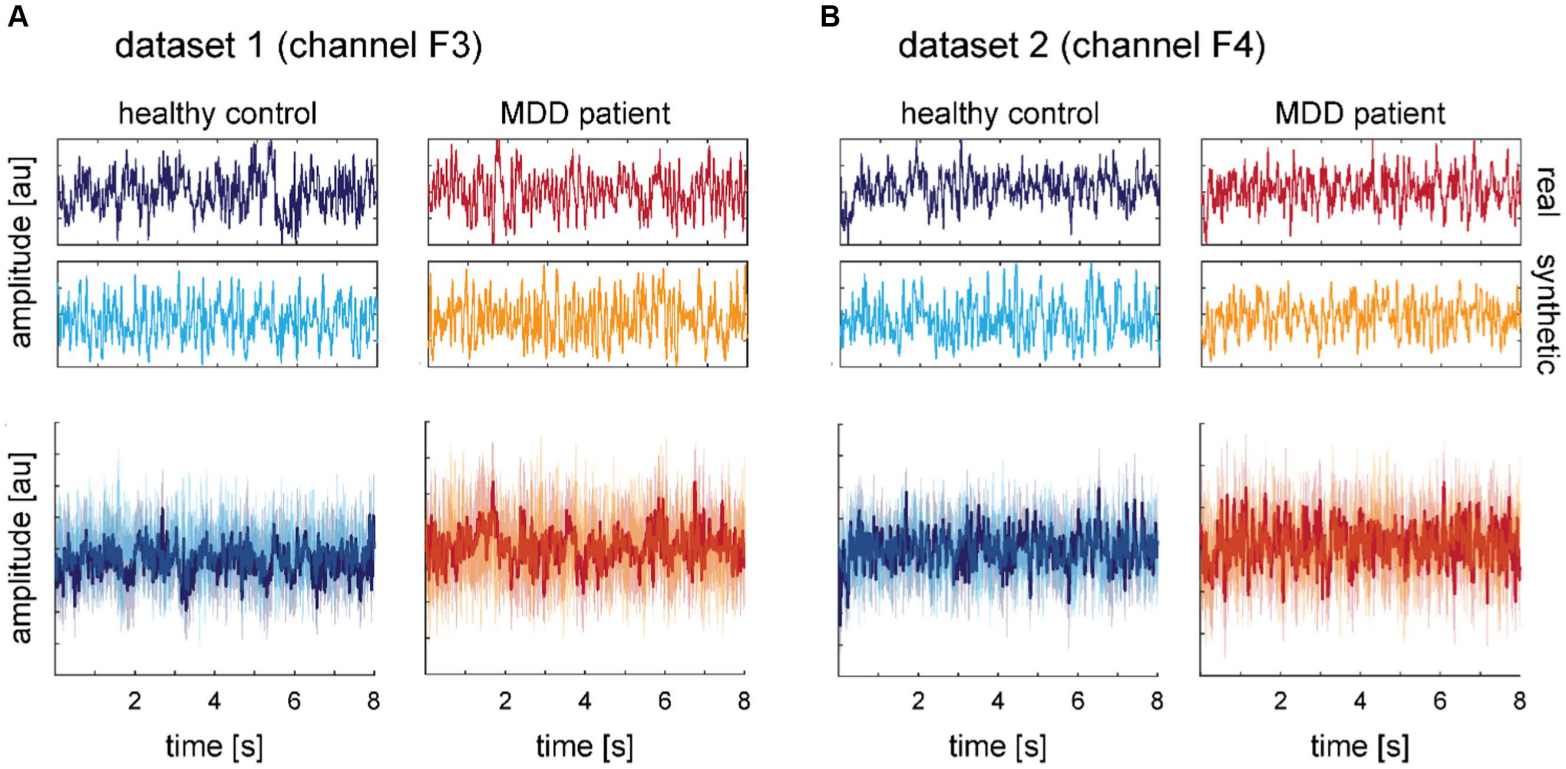
Comparison of time-series windows of 8 s in length for real and synthetic data for electrode F3 from dataset 1 **(A)** and electrode F4 from dataset 2 **(B)**. The top panels show exemplary single time-series for a random subject. Middle panels show exemplary single time-series for a random synthetic data frame. Bottom panels show the mean over 10 time-series from real subjects selected randomly and 10 time-series of synthetic data. The shaded areas depict 95% confidence intervals. Data is normalized from −1 to 1, therefore the amplitude has arbitrary units but y-axes match across graphs. The color scheme introduced in this figure is adopted in all subsequent figures showing real data (dark blue and red for HC and MDD, respectively) and synthetic data (light blue and orange for HC and MDD, respectively).

Visual inspection of exemplary single time-series (Figure 8 top rows) and means across time-series from electrodes F3 and F4 (Figure 8 bottom row) revealed no conspicuous differences between real and synthetic data. The forms and distributions of the generated time-series are well within the range expected from the real data.

**Figure 9:**
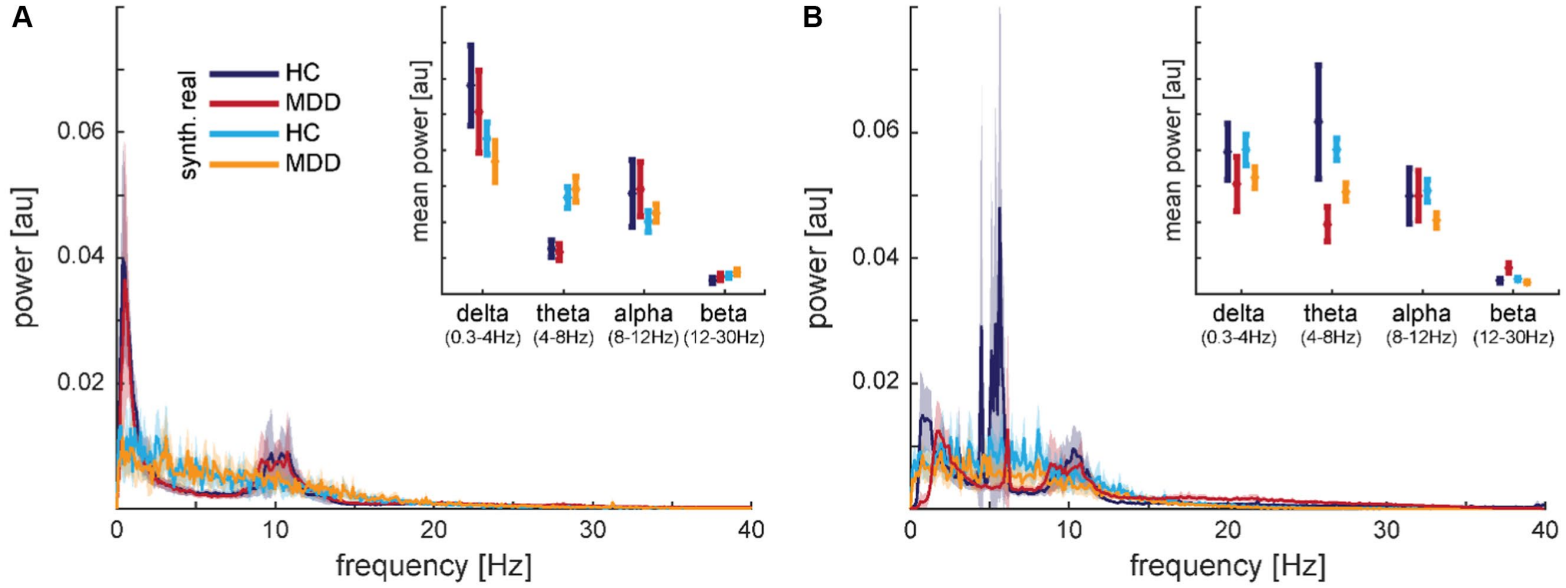
Comparison of mean spectra for real and synthetic data for electrode F7 from dataset 1 **(A)** and electrode F8 from dataset 2 **(B)**. The insets show the same data averaged within frequency bands. The shaded areas and error bars depict 95% confidence intervals. Data is normalized from −1 to 1, therefore, the amplitude has arbitrary units, but y-axes match across graphs.

Transforming the time-series to power spectra revealed that the synthetic data capture some aspects of the frequency content well but some aspects less well (*cf.* Figure 9 for electrodes F7 and F8). Well represented was the finding that most of the signal power is contained in frequencies roughly below 15 Hz. The characteristics in the low-frequency bands with peaks in delta and alpha but a dip in theta (except for some HC subject in dataset 2) bands seemed to be smoothed in the synthetic signals. Averaging the spectral power within frequency bands (*cf.* insets in Figure 9) revealed for dataset 1 significant main effects of data type, i.e., real vs. synthetic data, in delta, theta, and alpha bands (all *F_1,92_* > 5.041; *p* < 0.027) but neither main effects of diagnosis nor interactions (Figure 9A inset). Dataset 2 presented the opposite pattern, with significant main effects of diagnosis in delta and theta bands (all *F*_1,108_ > 7.389; *p* < 0.008) but neither main effects of data type nor interactions (Figure 9B inset). In the alpha band, we found no significant effects in the latter dataset.

In contrast to the synthetic data, we did not find any differences between real and noise data in the frequency bands. All main effects of data type failed to reach significance for dataset 1 (all *F*_1,92_ < 2.948; *p* > 0.089) and dataset 2 (all *F*_1,108_ < 2.251; *p* > 0.137).

#### 3.2.2. Classification results

**Figure 10:**
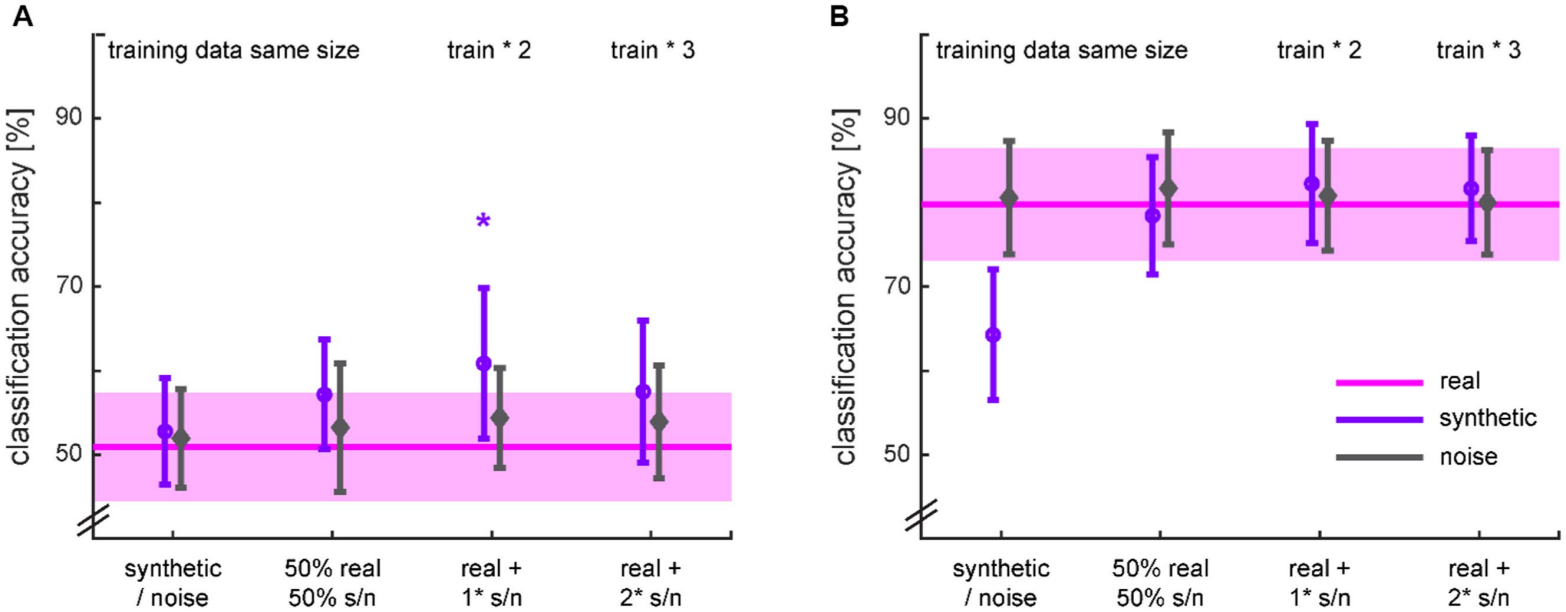
Classification accuracies for HC vs. MDD based on different ratios of real and synthetic or noise data for training (Table 2) from dataset 1 **(A)** and dataset 2 **(B)**. The classification with training on real data serves as baseline for comparison and is therefore spread across the x-axis. The shaded areas and error bars depict 95% confidence intervals. * *p* < 0.05 for the t-test in comparison to training on real data only.

The diagnosis classifiers trained on the real data performed around chance level (accuracy: 50.9 ± 6.4%) for dataset 1 and well above chance level (accuracy: 79.8 ± 6.7%) for dataset 2 (Figure 10 pink lines). The classifiers trained on the noise data performed in the same range, independent of the augmentation ratio. The classifiers trained only on the synthetic data performed either similar in the case of the chance classifier for dataset 1, or worse in the case of the performant classifier for dataset 2. In the latter case, however, it performed still significantly above chance level (*t_27_* = 16.168; *p* < 0.001). Substituting half of the real data by synthetic data brought back the performance of the original classifier for both datasets. Padding the real data with the same amount of synthetic data and therewith doubling the amount of training data yielded a significant accuracy increase of 9.96% in the case of dataset 1 (*t_46_* = 1.771; *p* = 0.042) but no significant improvement for dataset 2. Further adding synthetic data did not lead to additional improvements in classification accuracy. Note that the classifiers were all **tested** on real data.

Investigating the other classification performance metrics (Figure A1 in Supplementary material) indicated for dataset 1 that the classification was rather balanced for the real data. The increase in classification accuracy was first driven by the precision, but with the amount of real data being maximal again, the classification was balanced once more. The classification for dataset 2 was already tipped toward recall, i.e., sensitivity, for the real data and this imbalance was particularly pronounced when the classifier was exclusively trained by the synthetic data. However, augmenting the real data with twice the synthetic or noise data, balanced this classification as well.

## 4. Discussion

The current study presents the status of the field of generative methods for EEG data focusing on the generation of synthetic data later used for a classification task, such as a clinical diagnosis, based on a systematic review. An in-depth analysis of the methods, opportunities, and pitfalls, as well as the interdependence between the sub-steps of the data generation and evaluation process, provides an overview of the possibilities and current weaknesses of the research field. Based on two EEG datasets, we then demonstrated the generation of synthetic data from random noise for the two clinical groups MDD and HC with a WGAN incorporating CNNs as the generator and critic. The data were used to train a “diagnosis classifier” based on a CNN separating HC from MDD and were partially able to improve classification accuracy. This is to our knowledge the first study generating EEG time-series data directly for two clinical populations. The evaluation of our synthetic data reveals strengths and weaknesses of the generated data that are well within the parameters of comparable studies presented in the review part. The methods may not be ripe yet to be applied in neuroscience or medical research at a large scale to produce data for methodological developments. However, the field will now benefit greatly from domain experts working with EEG on understanding psychiatric or neurodegenerative diseases.

Evaluation of our synthetic data first demonstrates the face validity of the generated signal. The synthetic time-series cannot be distinguished from the real time-series with the bare eye. Transforming the data into the frequency space, a common transformation for extracting features from EEG signals (Poil et al., 2013), e.g., in order to extract biomarkers for MDD (Greco et al., 2021), revealed some weaknesses of our synthetic data. The generated data maps some frequency characteristics of the real data well but also smooths frequency peaks and dips. This effect can also be observed in the two other studies generating continuous time-series EEG data and subsequently showing frequency spectra. A close look at Figure 6 in the study of Hartmann et al. (2018) shows that, e.g., the beta peaks in the Rest condition are smoothed over in the synthetic data generated with a WGAN-GP with CNN architecture. Similarly, Figure 5 in the study of Bird et al. (2021) demonstrates highly smoothed versions of the 50 Hz line noise artifact as well as the absence of alpha peaks in the synthetic data produced with a GPT model. Given the importance of the frequency content in EEG signals, especially in clinical use cases (Poil et al., 2013), this constitutes a serious weakness in current generative models that needs to be addressed in further studies. Categorizing our power spectra in the frequency bands used in clinical research provides contradictory results. The analyses for dataset 1 showed that the real and synthetic data were significantly different in all frequency bands. However, for dataset 2, we found significant differences for the MDD vs. HC groups in the low frequency bands without significant differences between real and synthetic data. This finding demonstrates a successful differentiated reconstruction of this frequency content for the two clinical groups. This holds at least true for the granularity the frequency content is often analyzed, i.e. condensed in commonly used frequency bands. Note that for this dataset, the classification accuracy based on the real data is with nearly 80% already well above chance level. This suggests that the conditional GAN might also have a better chance of generating separable classes for a dataset in which the classes are already better separable. Finally, yet importantly, we improved our diagnosis classifier by nearly 10% for dataset 1 when augmenting the real data with the same amount of synthetic data. This is well within the range of improvement we saw in the other studies analyzed for the review. This improvement is mainly driven by precision, i.e., an increase in the ratio of correctly classified patients among all data classified as patients. It might be that the diversity of the augmented training data was helpful in this case. However, we could not replicate this improvement with dataset 2, which already had a marked higher baseline classification accuracy than dataset 1. Here, training only on synthetic data steeply increased the imbalance biased toward recall, i.e., the sensitivity or the ratio of correctly classified patients, at the cost of precision and specificity. Augmenting the data with synthetic or noise data, however, lead to a more balanced classification without increasing the classification accuracy. Because the CNN used for classification behaves like a black box, we are blind as to whether the algorithm bases its decision on clinical meaningful features or not. The direct comparison of the synthetic data as well as the classification results for the two datasets demonstrates that these methods do not yet produce stable results and therefore cannot be readily applied in a clinical context.

Our clinical use case of generating data for two clinical populations has only been studied twice, but both times with generating features from EEG instead of generating time-series data directly. An additional clinical study published after our search generated data for the minority class only (Sadegh-Zadeh et al., 2023). In contrast to within-subjects studies, like most other studies in the review, the differences between the classes arise here from disease-dependent changes to the resting state EEG signal and not from different mental states within the same subject. The inherent challenges of generating data resembling the original data persist, but the differentiation between classes is more subtle. In contrast to continuous EEG data, time locked signals are much more ordered in the time domain. Therefore, transferring methods between those two modes of EEG recordings should be considered with caution.

The type of generated data determines the degree of freedom for further processing the data. The less the EEG data is processed or condensed, the more options remain. The original EEG time-series can be reconstructed from a complex power spectrum, i.e., the amplitude and phase of the frequencies; therefore, this representation can also be used for obtaining data with the same possibilities as generating time-series data directly. Only one study used the real part of the power spectrum as input/output feature (Piplani et al., 2018), and four studies used images with TFR, i.e., spectral power amplitude over time. These representations do not allow for a full reconstruction of the signal since phase shifts between frequencies are lost, but the frequency content is still captured. Two of these studies presented exemplary synthetic TFR images (Zhang and Liu, 2018; Zhao et al., 2020) that seem to capture the frequencies rather well, but the overall quality of the generated data cannot be fully judged based on these images alone. Apart from data type, the structure of the input data is of relevance, at least for the generators using convolutional layers. Here, the neighborhood relations in the data structure are of essence. For time and frequency dimensions, these relationships are given but the spatial domain, i.e., the channels, were handled differently across studies. Choosing only one channel (Hartmann et al., 2018) or separately generating data for each channel (Zhang and Liu, 2018) bypasses this issue. However, the signal correlation between channels is discarded in the synthetic data in the latter case. Zhang et al. (2020) stacked the TFR images in the same dimension as the frequency while maintaining the neighboring relationships between their three channels. Several studies organized the channels in an additional dimension but did not report their order. Finally, the type of normalization of the EEG time-series is important to consider, especially when the synthetic dataset is supposed to be used for a clinical use case. Normalization from zero to one or minus one to one is a common preprocessing step for machine learning (Singh and Singh, 2020), i.e., also for the generator models. For many (clinical) applications, however, the relative signal strength across electrodes is meaningful as demonstrated by the common representation of EEG data in a topographic map. These differences should therefore not be factored out by, e.g., normalizing the channels individually. The same holds true for other common preprocessing steps which are out of scope for this review but can heavily influence further processing and should therefore be carefully chosen based on domain expertise. Domain knowledge of the use case and the data also aids in extracting features from the EEG data and generating those instead of time-series data. This is a viable option when the data is used directly to augment the training data for a specific classification task. However, this approach limits the use of the synthetic data beyond this immediate application.

For GANs, various architectural choices have already been tested for generating EEG data (*cf.* Figure 3). This toolbox provides a solid foundation for refining the models for generating EEG data usable for a clinical use case. Especially for time-series transformed into TFR images, CNN variants of the GAN provide the advantage of a large community working on image generation and with it the quick advances in methodological development (Wang et al., 2021). However, the issue of a potential spatial dimension with the EEG channels still needs to be addressed. Given the sequential nature of EEG signals, GAN architectures using RNN and GPT models seem to be a natural choice. However, these approaches have been studied less frequently, and their potential for the clinical use case needs more exploration. In light of the rapidly advancing fields of language and image generation (Zhang et al., 2023), researchers synthetizing EEG data should keep track of GPT and possible future classes of generative algorithms. Except for one study, VAE were only used for comparison with a GAN architecture and always performed worse than the GAN.

A third of the studies provided neither a qualitative nor a quantitative evaluation of the quality of the generated data. Given that data quality is of essence for any data-driven decision process, selective and meaningful metrics for assessing data quality, and in the case of synthetic data, faithfulness to the real data, are essential. Evaluation metrics for synthetic data are still a topic of ongoing research and debate (Theis et al., 2015; Borji, 2019). Face validity is a first important step. However, in the case of EEG time-series data, which cannot be judged as easily as, e.g., natural images by the naked eye, showing time-series data is not sufficient. Transformation into frequency space or visual representations like topographic maps aids visual judgment tremendously and does in fact reveal weaknesses of the synthetic data in the few studies that provided this information as well as in our own. Finding two- or three-dimensional mappings of the data that represent its distribution are popular methods that were also applied in some of the reviewed studies. However, the three studies that did show convincing 2D distributions of the time-series data (Fahimi et al., 2021; Geng and Chen, 2021; Xu et al., 2022) all worked with time locked data, e.g., the form of the ERP was represented in 2D space and not a continuous EEG signal. The only study showing a 2D representation of continuous time-series data with a principal component analysis (PCA) (Kalashami et al., 2022) could neither demonstrate a class nor dataset separation in this representation. We also tried to find a meaningful 2D representation with three popular dimension reduction approaches—PCA, locally linear embedding [LLE (Roweis and Saul, 2000)], and t-Distributed Stochastic Neighbor Embedding [t-SNE (Van der Maaten and Hinton, 2008)]—but failed to find one. Explaining only 8 to 9% of the variance in our data with the first two components of the PCA suggests that two dimensions might simply not suffice for meaningfully condensing continuous time-series EEG data. Quantitative metrics were provided by seven studies only and the details of the evaluation vary widely. For time locked paradigms, similarity between signals can be assessed more easily, e.g., DTW (Bellman and Kalaba, 1959) is a distance metric applied in one study that is well suited for a comparison between two time-series with defined beginning and ending. For continuous data, however, quantitative evaluation of the data quality might greatly benefit from domain expertise about the use case. In our study, we extracted the bandpower of the available frequency bands for a quantitative evaluation. These features, however, are only one example of the many biomarkers used in EEG research for MDD (Greco et al., 2021).

The gold standard evaluation of the synthetic data for most studies was improving a classifier’s performance with training it on augmented data including the synthetic data. All studies succeeded in improving their classification accuracy. However, 18 studies used some kind of deep learning classifier and did not assess whether the synthetic data genuinely provided relevant information for the task or whether accuracy improvement was simply an artifact (Nguyen et al., 2015). An explanation for the pattern of only a transient accuracy increase with increasing amount of synthetic data observed in a few studies might be that the first couple of data frames counteract the effect of overtraining but further synthetic data tunes the classifier too far away from using task-relevant information. This hypothesis, however, is of a theoretical nature so far and needs further investigation. In our work, we compared the performance of a classifier trained on data augmented with synthetic data to one trained on data augmented with noise data. While adding noise data did not improve the classification performance significantly, adding synthetic data did in the case of dataset 1. However, we classified the data with a CNN used as a black box starting at the chance level. For the classifier starting well above chance level with dataset 2, we could not replicate this improvement.

The articles reviewed were mainly directed at a technical or methodologically interested and adept audience. Algorithms were developed or adapted from another data domain, and the main goal was to demonstrate the technical feasibility. In order to proceed to the generation of synthetic data with clinical relevance, i.e., faithfully representing clinically relevant features in the data, domain experts on EEG data analysis in a clinical area or in basic neuroscience research need to add their expertise to the research field. This review has identified two key issues where domain expertise is essential: the format of the input/output data and evaluation of the generated data. On a related note, the explainability of DL models processing EEG data needs to be enhanced, another task where domain expertise is most useful. This becomes relevant to the field when the DL model is used to evaluate the generated data. Finally, yet importantly, architectures specifically designed for continuous data such as GANs with RNN and GPT models should be further explored for their suitability for EEG data generation.

Our work carves out the opportunities and current weaknesses of generating synthetic EEG data for two clinical groups, such as MDD patients and HC, based on a systematic literature review in combination with an empirical study on two publicly available datasets. The generation of synthetic data constitutes a promising approach for (medical) fields in which large datasets are sparse. Still, biomarker research, especially methods based on (deep) machine learning, requires large datasets to produce generalizable models able to support clinical routine. A sound technical basis is set with the algorithms developed over the last decade, but the shortcomings of the data generated so far require further research before their broad application in clinical use cases. In order to address these shortcomings, more domain expertise from researchers specialized in EEG processing and EEG biomarkers for clinical applications needs to be incorporated into further developments in the field.

## Data availability statement

Publicly available datasets were analyzed in this study. This data can be found here: http://modma.lzu.edu.cn/data/index/; https://doi.org/10.6084/m9.figshare.4244171.v2.

## Ethics statement

The studies involving humans were approved by Ethics Committee for Biomedical Research at the Lanzhou University Second Hospital and Ethics Committee of Hospital Universiti Sains Malaysia. The studies were conducted in accordance with the local legislation and institutional requirements. The participants provided their written informed consent to participate in this study.

## Author contributions

YH organized and pre-processed the data. FC performed data augmentation and classification. FC and AR analyzed the results and performed the literature review. AR wrote the first draft of the manuscript. FC and YH wrote sections of the manuscript. All authors contributed to conception and design of the study. All authors contributed to manuscript revision, read, and approved the submitted version.
