# Supplementary material for: Generation of synthetic EEG data for training algorithms supporting the diagnosis of major depressive disorder
Source: Front Neurosci. 2023 Oct 2;17:1219133. doi: 10.3389/fnins.2023.1219133 (PMC10577178; doi:10.3389/fnins.2023.1219133)
Supplement: Supplementary file 1 [file Table_1.pdf]

Appendix

Table A1 Architecture of the generator for dataset 2. The generator for dataset 1 is constructed accordingly with adjustments to the size of the real data (2000 instead of 2048 data points for the time dimension). Orange: latent input; blue: label information.

Layer	Output Shape	Param #	Kernel Shape	Stride
Input Layer	(100)	0		
Input Layer	(1)	0		
Dense	(32768)	3,309,568		
Embedding	(1, 50)	100		
Leaky ReLU	(32768)	0		
Dense	(1, 256)	13,056		
Reshape	(1, 256, 128)	0		
Reshape	(1, 256, 1)	0		
Concatenate	(1, 256, 129)	0		
Conv2D Transpose	(2, 512, 128)	148,736		
Batch Normalization	(2, 512, 128)	512		
Leaky ReLU	(2, 512, 128)	0		
Conv2D Transpose	(4, 1024, 128)	147,584	(3,3)	(2,2)
Batch Normalization	(4, 1024, 128)	512		
Leaky ReLU	(4, 1024, 128)	0		
Conv2D Transpose	(8, 2048, 128)	147,584	(3,3)	(2,2)
Batch Normalization	(8, 2048, 128)	512		
Leaky ReLU	(8, 2048, 128)	0		
Conv2D Transpose	(16, 2048, 128)	147,584	(3,3)	(2,1)
Batch Normalization	(16, 2048, 128)	512		
Leaky ReLU	(16, 2048, 128)	0		
Conv2D	(13, 2048, 32)	16,416	(4,1)	(1,1)
Batch Normalization	(13, 2048, 32)	128		
Leaky ReLU	(13, 2048, 32)	0		
Conv2D	(13, 2048, 1)	801	(5,5)	(1,1)

Total params: 3,933,605

Trainable params: 3,932,517

Non-trainable params: 1,088

Table A2 Architecture of the critic for dataset 2. The critic for dataset 1 is constructed accordingly with adjustments to the size of the real data (2000 instead of 2048 data points for the time dimension). Orange: latent input; blue: label information.

Layer	Output Shape	Param #	Kernel Shape	Stride
Input Layer	(1)	0		
Embedding	(1, 50)	100		
Dense	(1, 26624)	1,357,824		
Input Layer	(13, 2048, 1)	0		
Reshape	(13, 2048, 1)	0		
Concatenate	(13, 2048, 2)	0		
Gaussian Noise	(13, 2048, 2)	0		
Conv2D	(7, 1024, 128)	2,432	(3,3)	(2,2)
Leaky ReLU	(7, 1024, 128)	0		
Conv2D	(4, 512, 128)	147,584	(3,3)	(2,2)
Leaky ReLU	(4, 512, 128)	0		
Conv2D	(4, 256, 128)	147,584	(3,3)	(1,2)
Leaky ReLU	(4, 256, 128)	0		
Flatten	(131072)	0		
Dropout	(131072)	0		
Dense	(1)	131,073		
Total params: 1,786,597				
Trainable params: 1,786,597				
Non-trainable params: 0				

This is a provisional file, not the final typeset article

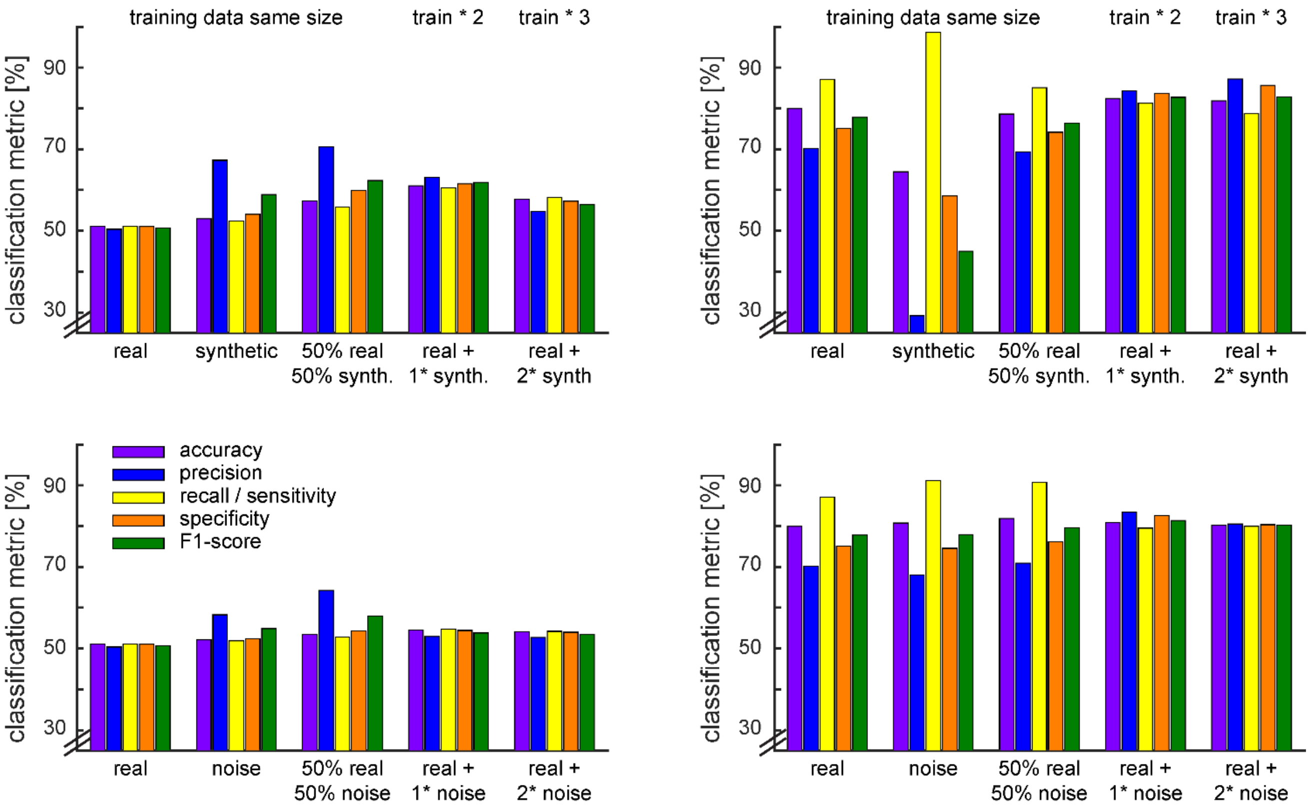

Figure A1 Classification performance metrics for HC *vs.* MDD based on different ratios of real and synthetic (upper row) or noise data (lower row) for training (Table 2) from dataset 1 (left column) and dataset 2 (right column).
