# Supplementary figures and images for: Generation of synthetic EEG data for training algorithms supporting the diagnosis of major depressive disorder
Source: Front Neurosci. 2023 Oct 2;17:1219133. doi: 10.3389/fnins.2023.1219133 (PMC10577178; doi:10.3389/fnins.2023.1219133)

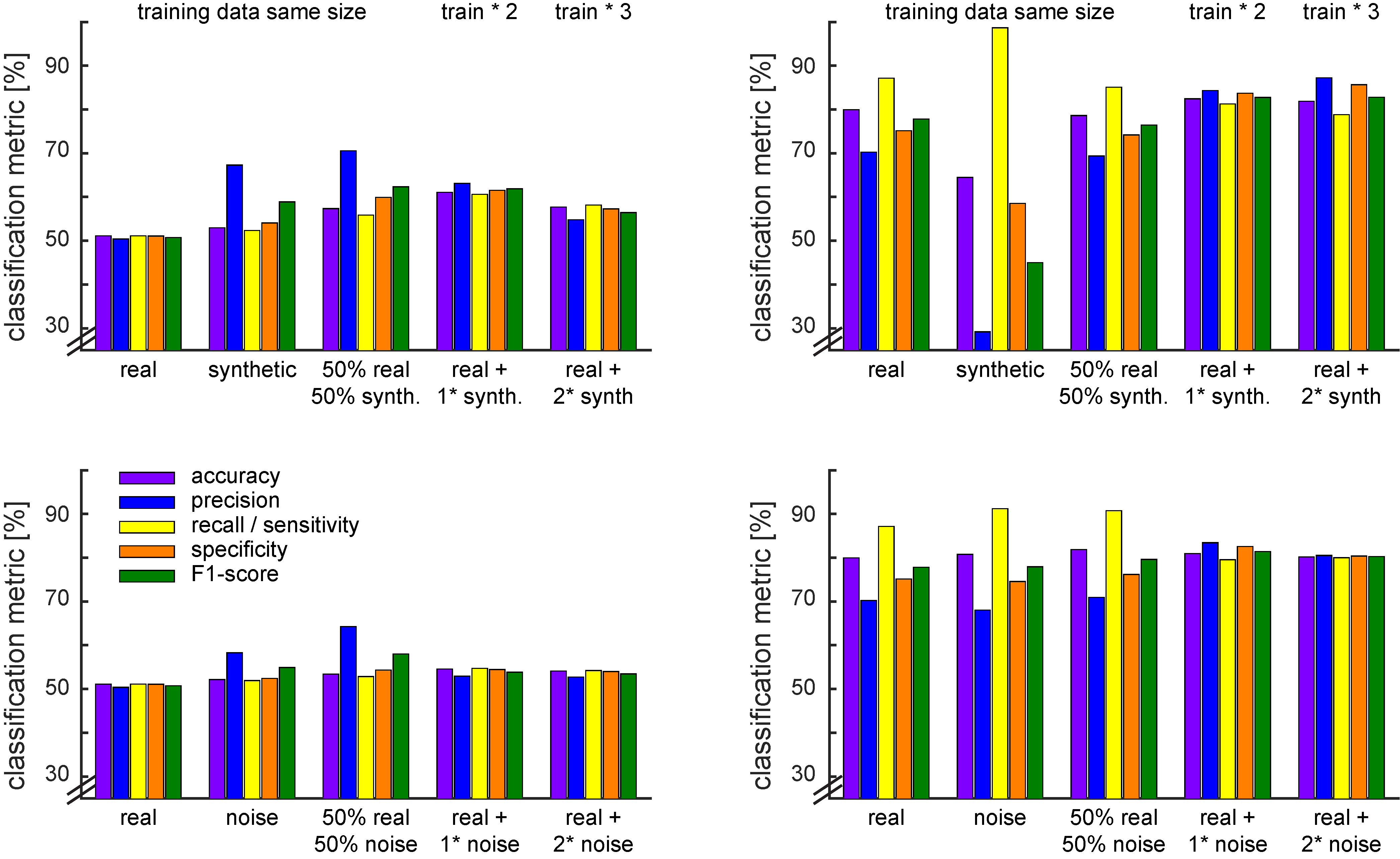

Supplement: Supplementary file 2 [file Image_1.TIF]
